# Supplementary material for: High-throughput mutagenesis reveals unique structural features of human ADAR1
Source: Nat Commun. 2020 Oct 12;11:5130. doi: 10.1038/s41467-020-18862-2 (PMC7550611; doi:10.1038/s41467-020-18862-2)
Supplement: Supplementary file 1 — Supplementary Information [file 41467_2020_18862_MOESM1_ESM.pdf]

Supplementary Information

High-throughput Mutagenesis Reveals Unique Structural Features of Human ADAR1

Park *et al.*

```
                                     α1                    β1           β2
ADAR1 H. sapiens      LPLTGSTFHDQIAMLSHRCFNTLTNSFQPSLLGRKILAAIIMKKDSEDMG.VVVSLGTGNRCVKGDSLS 900
ADAR1 R. norvegicus   LPLTGSTFHDQIAMLSHRCFNALTNSFQPSLLGRKILAAIIMKRDPEDMG.VVVSLGTGNRCVKGDSLS 846
ADAR1 M. musculus     LPLSGSTFHDQIAMLSHRCFNALTNSFQPSLLGRKILAAIIMKRDPEDMG.VVVSLGTGNRCVKGDSLS 849
ADAR1 M. mulatta      LPLTGSTFHDQIAMLSHRCFNTLTNSFQPSLLGRKILAAIVMKKDSEDMG.VVVSLGTGNRCVKGDSLS 899
ADAR1 T. rubripes     LPVTGSTLHDQIAMLSHQRFNALTTRIQHSLLGRKILATIIMRKG-DGLG.TVVSLGTGNRCVKGEELS 981
ADAR1 G. gallus       LPVRGSTLHDQMAMLSHQRFNVLTACIQHSLLGRKILAAIIMRKGSQDLG.VVVSIGTGNRCVKGEELS 525
ADAR1 D. rerio        LPVSGSTIHDQIAMLSHQRFNALTARIQHSLLGRKILATIVMRNGTDSLG.KVVSLGTGNRCVKGEELS 1018
ADAR1 X. laevis       LPVSGSTFHDQIAMLSHQKFNSLTARIQNSLLGRKILAAIIMKKSSDDLG.TVVSIGTGNRCVKGEELS 946
ADAR2 H. sapiens      QLHLPQVLADAVSRLVLGKFGDLTDNFSSPHARRKVLAGVVMTTGTDVKDAKVISVSTGTKCINGEYMS 384
ADAR2 R. norvegicus   QLHLPQVLADAVSRLVLGKFSDLTDNFSSPHARRKVLSGVVMTTGTDVKDAKVISVSTGTKCINGEYMS 384

                                     α2           α3    β3    β4       β5
ADAR1 H. sapiens      LKGETVNDCHAEIISRRGFIRFLYSELMKYNSQ...TAKDSIFEP.AKGGEKLQIKKTVSFHLYISTAP 965
ADAR1 R. norvegicus   LKGETVNDCHAEIISRRGFIRFLYSELMKYNHH...TAKNSIFEL.ARGGEKLQIKKTVSFHLYISTAP 911
ADAR1 M. musculus     LKGETVNDCHAEIISRRGFIRFLYSELMKYNHH...TAKNSIFEL.ARGGEKLQIKKTVSFHLYISTAP 914
ADAR1 M. mulatta      LKGETVNDCHAEIISRRGFIRFLYSELMKYNPQ...TAKDSIFEP.AKGGEKLQIKKTVSFHLYISTAP 964
ADAR1 T. rubripes     LKGDTVNDCHAEIISRRGFVRFVYSEILKYQDG....NDDCIFEP.AE.NNKLQVKPDTTFHLYISTAP 1044
ADAR1 G. gallus       LKGETVNDCHAEIISRRGFVRFLYSELMKYDPCKPS.AEESIFEP.AG.GRRLKIKSSITFHLYISTAP 591
ADAR1 D. rerio        LRGDTVNDCHAEIISRRGFIRFLYSELMKHWES...PGDETIFEL.AG.DGKLKIKSDITFHLYISTAP 1082
ADAR1 X. laevis       LRGETVNDCHAEIVSRRGFIRFLYSQLMKYNPD...VPGDSIFEE.AD.GDLLRVRPGVTFHLYISTAP 1010
ADAR2 H. sapiens      DRGLALNDCHAEIISRRSLLRFLYTQLELYLNNKDD.QKESIFQK.SE.RGGFRLKENVQFHLYISTSP 450
ADAR2 R. norvegicus   DRGLALNDCHAEIISRRSLLRFLYAQLELYLNNKED.QKKSIFQK.SE.RGGFRLKDTVQFHLYISTSP 450

                              5' binding loop       β6                   α4     β7
ADAR1 H. sapiens      CGDGALFDKSCSDRAMESTESRHYPVFENPKQGKLRTKVENGEGTIPVESSDIVPTWDGIRLGERLRTM 1034
ADAR1 R. norvegicus   CGDGALFDKSCSDRAVESTESRHYPVFENPKQGKLRTKVENGEGTIPVESSDIVPTWDGIRLGERLRTM 980
ADAR1 M. musculus     CGDGALFDKSCSDRAVESTESRHYPVFENPKQGKLRTKVENGEGTIPVESSDIVPTWDGIRLGERLRTM 983
ADAR1 M. mulatta      CGDGALFDKSCSDRAMESTDSRHYPVFENPKQGKLRTKVENGEGTIPVESSDIVPTWDGIRLGERLRTM 1033
ADAR1 T. rubripes     CGDGALFDKSCSETGDEI..KGHQPLFENVKQGKLRTKVENGEGTIPVESSAIVPTWDGIQHGERLRTM 1111
ADAR1 G. gallus       CGDGALFDKSCSDQASVVGPAEHQPLFENSKQGKLRTKVENGEGTIPVESSDIVPTWDGIQHGERLRTM 660
ADAR1 D. rerio        CGDGALFDKSCSEAAE-LNGSGHMPLFENIKQGKLRTKVENGEGTIPVESSDIVPTWDGIQHGERLRTM 1150
ADAR1 X. laevis       CGDGALFDKSCSDQPSAEGDNKHCPIFENVKQGKLRTKVENGEGTIPVESSDIVPTWDGIQHGERLRTM 1079
ADAR2 H. sapiens      CGDARIFSPHEPILE.....EPADRHPNRKARGQLRTKIESGEGTIPVRSNASIQTWDGVLQGERLLTM 514
ADAR2 R. norvegicus   CGDARIFSPHEPVLE.....EPADRHPNRKARGQLRTKIESGEGTIPVRSNASIQTWDGVLQGERLLTM 514

                          α5        α6         β8          α7
ADAR1 H. sapiens      SCSDKILRWNVLGLQGALLTHFLQPIYLKSVTLGYLFSQGHLTRAICCRVTRDGSAFEDGLRHPFIVNH 1103
ADAR1 R. norvegicus   SCSDKILRWNVLGLQGALLTHFLQPVYLKSVTLGYLFSQGHLTRAICCRVTRDGNAFEDGLRYPFIVNH 1049
ADAR1 M. musculus     SCSDKILRWNVLGLQGALLTHFLQPVYLKSVTLGYLFSQGHLTRAICCRVTRDGKAFEDGLRYPFIVNH 1052
ADAR1 M. mulatta      SCSDKILRWNVLGLQGALLTHFLQPIYLKSVTLGYLFSQGHLTRAICCRVTRDGSAFEDGLRHPFIVNH 1102
ADAR1 T. rubripes     SCSDKILRWNVLGLQGALLSHFINPIYLKSITLGYLYSHGHLTRAVCCRMATNGQEFAQSLSAPFMLNH 1180
ADAR1 G. gallus       SCSDKILRWNVLGLQGALLSHFIEPVYLSSVTLGYLYSQGHLTRAICCRLVRDGDVLQKRLQAPYQINH 729
ADAR1 D. rerio        SCSDKILRWNVLGLQGALLTHFIHPIYLHSITLGYLYSHGHLTRAVCCRLSRDGDTFKSSLPANFTLNH 1219
ADAR1 X. laevis       SCSDKILRWNVLGLQGGLLTHFMEPVYLSSLTLGYLFSKGHLTRAICCRMSRDGDAFQNQLPDLYLVNH 1148
ADAR2 H. sapiens      SCSDKIARWNVVGIQGSLLSIFVEPIYFSSIILGSLYHGDHLSRAMYQRISNI.....EDLPPLYTLNK 578
ADAR2 R. norvegicus   SCSDKIARWNVVGIQGALLSIFVEPIYFSSIILGSLYHGDHLSRAMYQRISNI.....EDLPPLYTLNK 578

                       β9               β10        β11                 α8
ADAR1 H. sapiens      PKVGRVSIYDSKRQSGKTKETSVNWCLADGYDLEILDGTRGTVDGPRNELSRVSKKNIFLLFKKLCSF. 1171
ADAR1 R. norvegicus   PKVGRVSVYDSKRQSGKTKETSVNWCLADGYDLEILDGTRGTVDGPGKELSRVSKKNIFLQFKKLCSF. 1117
ADAR1 M. musculus     PKVGRVSVYDSKRQSGKTKETSVNWCMADGYDLEILDGTRGTVDGPGKELSRVSKKNIFLQFKKLCSF. 1120
ADAR1 M. mulatta      PKVGRVSVYDSKRQSGKTKETSVNWCLADGYDLEILDGTRGTVDGPRNELSRVSKKNIFLLFKKLCSF. 1170
ADAR1 T. rubripes     PEVGRVSVYDSTRHTGKTKESSVNWSFPDQHSVEVLDGTTGKLDGNKLSVSRVTKSNLFALFRAVCQR. 1248
ADAR1 G. gallus       PEVGRVSVYDSARQTGKTKESSVNWCLADGSKVEVLDGTKGKVDGPKLEVSRVSKRKTFALFQQLCAK. 797
ADAR1 D. rerio        PEVGRVSVYDSTRHTSKTKESSVNWSQPDQYSVEVLDGTKGKLDSPKMEVSRVSKSNLFRLFHALCQR. 1288
ADAR1 X. laevis       PEVGRVSVYDSTRQTGKTKESSVNWCLADE.EAEVLDGTKGKVEGAKLEISRVSKLHMFTLFQELCLL. 1215
ADAR2 H. sapiens      PLLSGISNA.EARQPGKAPNFSVNWTVGDS.AIEVINATTGKDELGR..ASRLCKHALYCRWMRVHGKV 643
ADAR2 R. norvegicus   PLLSGISNA.EARQPGKAPNFSVNWTVGDT.AIEVINATTGKDELGR..PSRLCKHALYCRWMRVHGKV 643

                                  α9           α10
ADAR1 H. sapiens      ...RYRRDLLRLS.YGEAKKAARDYETAKNYFKKGLKDMGYGNWISKPQEEKNFYLCPV 1226
ADAR1 R. norvegicus   ...RARRDLLQLS.YGEAKKAARDYDLAKNYFKKSLRDMGYGNWISKPQEEKNFYLCPV 1172
ADAR1 M. musculus     ...RARRDLLQLS.YGEAKKAARDYDLAKNYFKKSLRDMGYGNWISKPQEEKNFYLCPV 1175
ADAR1 M. mulatta      ...RYRRDLLRLS.YGEAKKAARDYETAKNYFKKSLKDMGYGNWISKPQEEKNFYLCPV 1225
ADAR1 T. rubripes     ...CGRTDLLSLHSYSQAKMAALSFQLAKQQFFQALTAHGYGTWIGKPLEEKSFEAGES 1304
ADAR1 G. gallus       ...SDCKELQKLSVYSEAKKAAVQYQKAKQCFFSALEEMGYGSWICKPQEEDNFSIPDA 853
ADAR1 D. rerio        ...AGRADLLALQSYAHAKMAATSFQEAKRLFFLALSQHGYGAWIGKPLEEKSFEGEAK 1343
ADAR1 X. laevis       ...RGRHDLLALSSYSDVKATVGTYQTARGQFFRALEQMGYGNWISKPQEEKCFSLSI. 1270
ADAR2 H. sapiens      PSHLLRSKITKPNVYHESKLAAKEYQAAKARLFTAFIKAGLGAWVEKPTEQDQFSLTP. 701
ADAR2 R. norvegicus   PPHLLRTKITKPTTYHESKLAAKEYQAAKARLFTAFIKAGLGAWVEKPTEQDQFSFTP. 701
```

Supplementary Figure 1. Sequence alignment of hADAR deaminase domains from various organisms with secondary structural elements observed in hADAR2d bound to dsRNA crystal structure [1,2]. Putative binding residues for the second zinc site within hADAR1d are shown in red and the 5' binding loop sequence is highlighted by salmon.

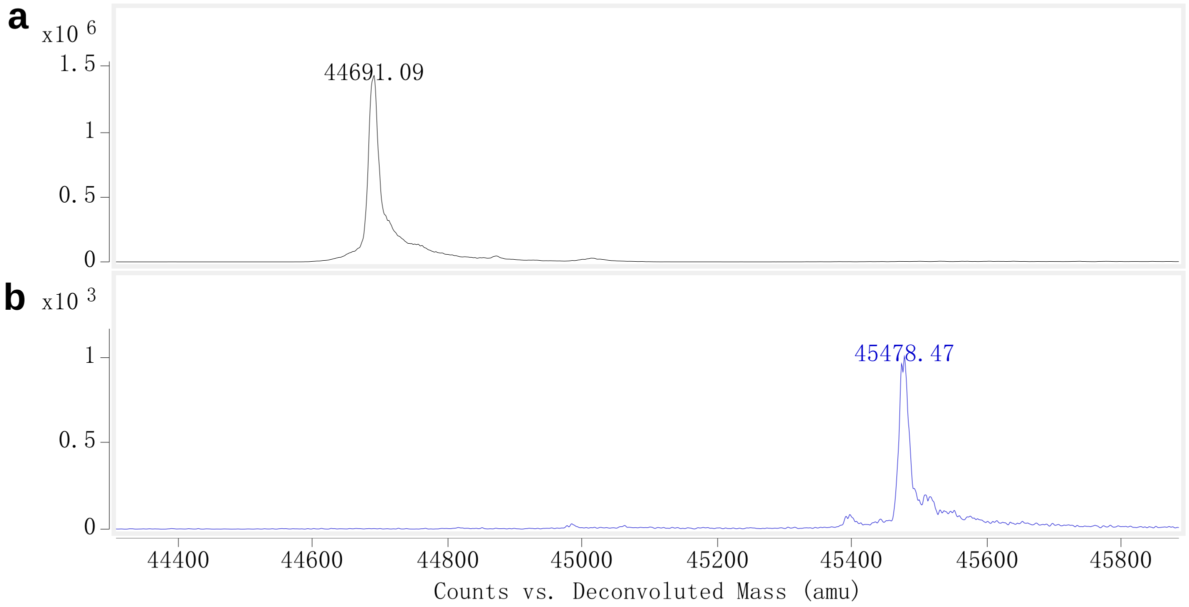

Supplementary Figure 2. Electrospray ionization-mass spectrometry analysis confirms the bound ligand composition in hADAR1d WT. (a) Experimental intact mass of hADAR1d WT matches with the calculated mass (44690.26 amu). (b) Native mass of hADAR1d WT show an addition of 787.38 amu corresponding to non-covalently bound IP_6 (659.86 amu) and two Zn^{+2} ions (130.76 amu).

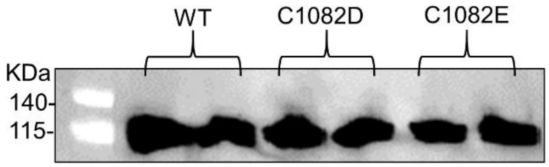

Supplementary Figure 3. Western blot of hADAR1 p110 WT, C1082D and C1082E in HEK293T cells. 750ng of each hADAR1 plasmids were transfected for 48 h in HEK293T cells, which then subsequently lysed for western blotting. Western blot analysis was conducted once using two different biological samples overexpressing each protein. Source data are provided in the Source Data file.

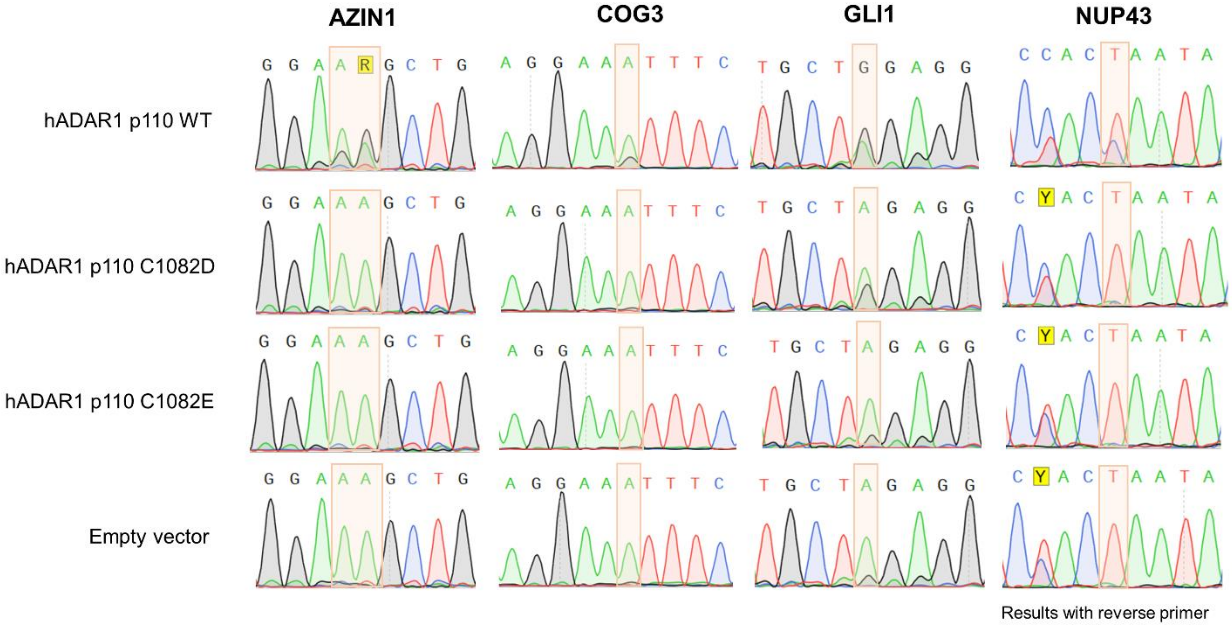

Supplementary Figure 4. Sequencing traces of endogenous target sites (AZIN1, COG3, GLI1, NUP43) in HEK293T cells edited by hADAR1 p110 WT, C1082D, C1082E and control empty vector.

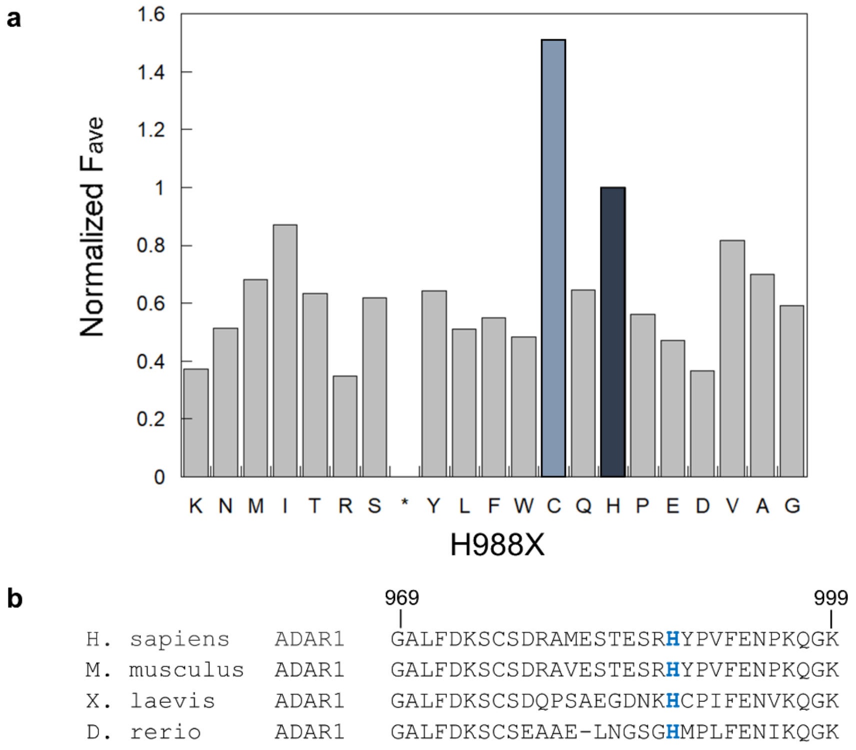

Supplementary Figure 5. Sat-FACS-seq screening result of hADAR1d H988 residue and sequence alignment of ADAR1 [3]. (a) H998 to C988 mutation showed the activated deaminase activity. (b) H988 (highlighted in blue) is conserved among other ADAR1 from different organisms, suggesting a biological importance of this residue.

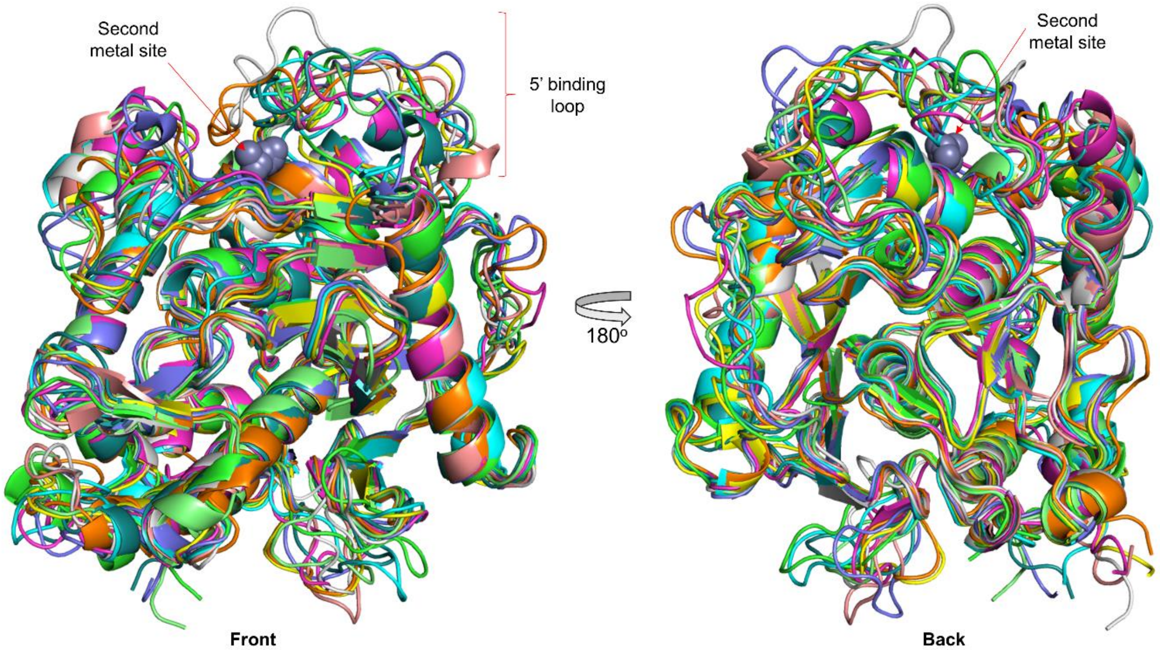

Supplementary Figure 6. An overlay of the top ten lowest energy structures generated by structural modeling. The 5' binding loop region shows divergent conformations, whereas most of core structures show a high convergence. All input files and the lowest 10 energy structures are available in GitHub at https://github.com/siegel-lab-ucd/Publication_Tiffy/tree/master/High-throughput%20Mutagenesis%20Reveals%20Unique%20Structural%20Features%20of%20Human%20ADAR1

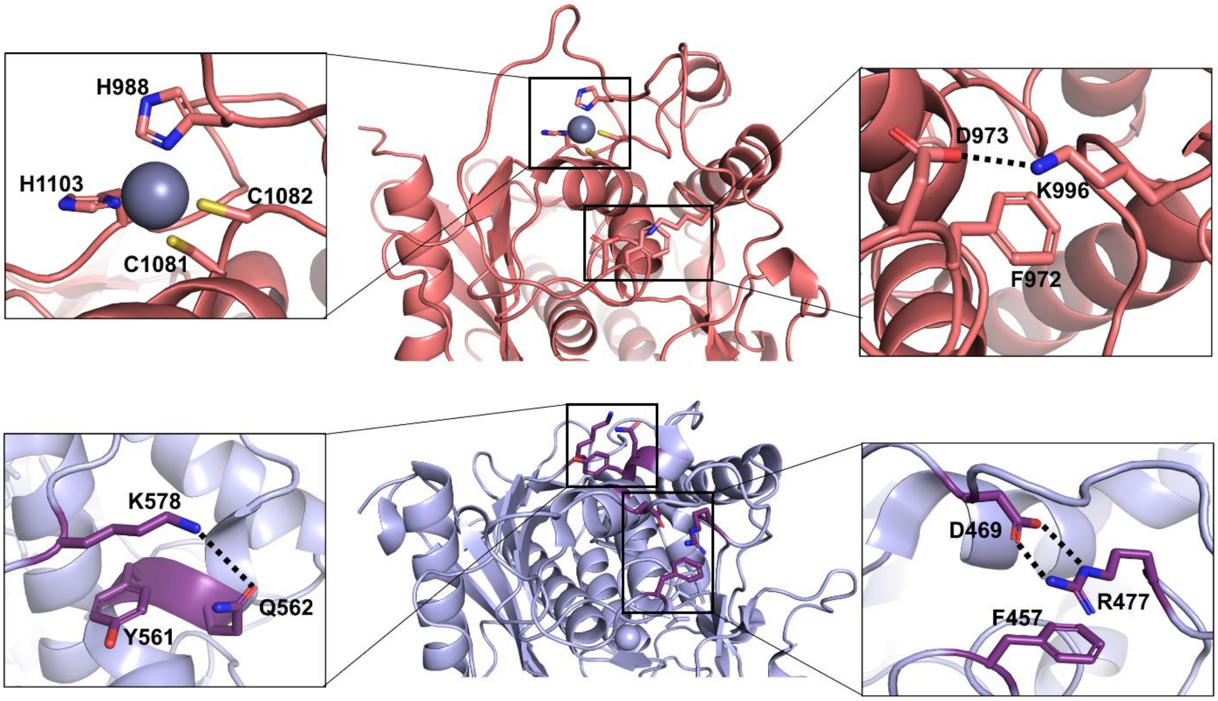

Supplementary Figure 7. Comparison of constraints used to generate structural models of ADAR1d (Top structure in Salmon) to high resolution structure of ADAR2d [1] (Bottom structure in Purple). (Right) Comparison of interactions of three residues that stabilize the 5' binding loop conformation. (Left) Comparison of the second metal binding site and corresponding residues in hADAR2d crystal structure.

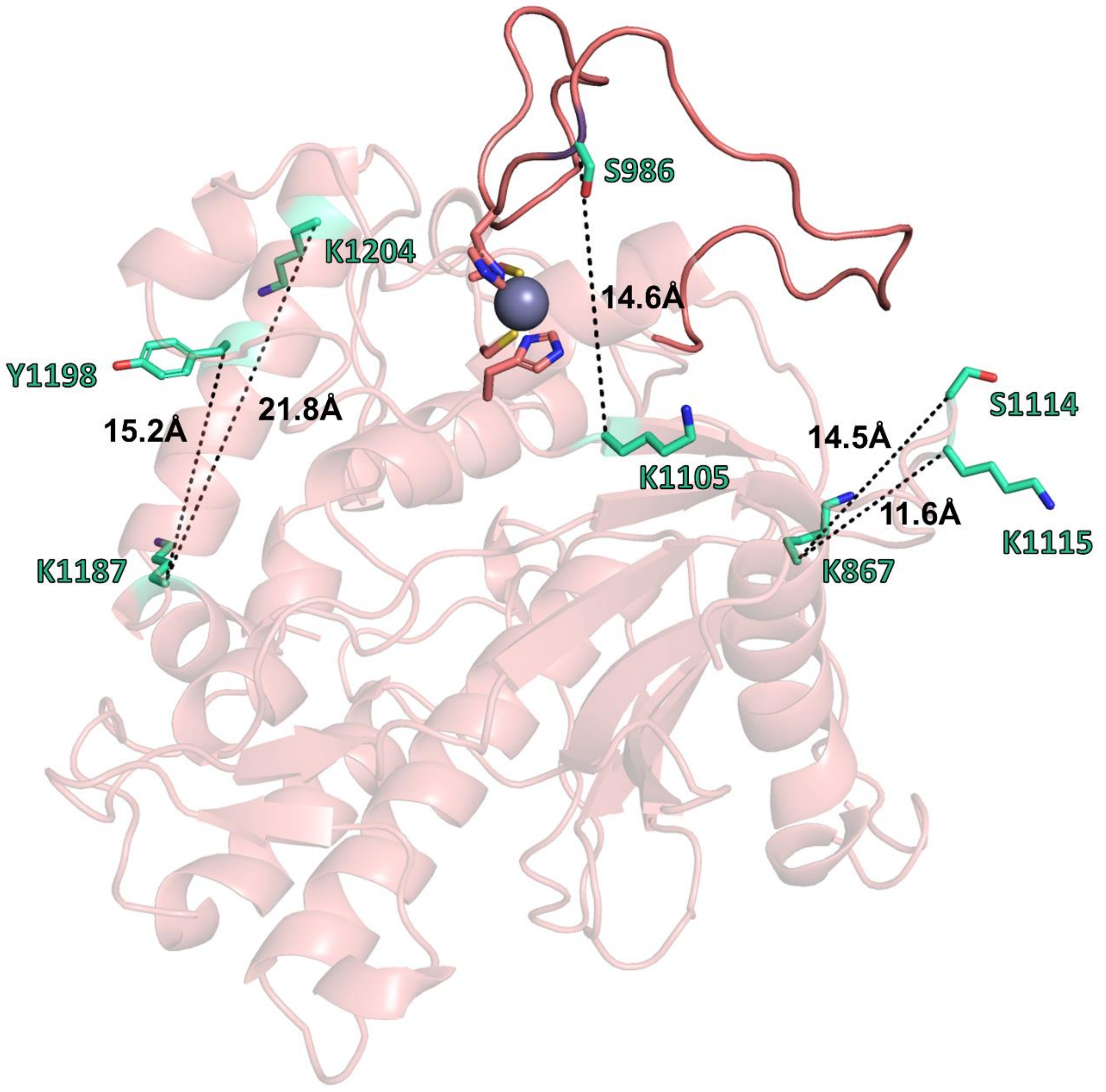

Supplementary Figure 8. Cross-linked residues identified in all three independent replicates by XL-MS using DSBU. Cross-linked residue pairs repeatedly identified from all three replicates are mapped into hADAR1d homology model. The second metal binding site and the 5' binding loop are shown in salmon and residues involved in cross-linking are represented in green cyan. A Cα-Cα distance (Å) for each cross-linking is shown in black dotted line.

Supplementary Figure 9a-e. Representative annotated MS2 spectra of cross-linked residues identified in all three independent replicates using DSBU. All spectrum is assigned by the MeroX software [4].

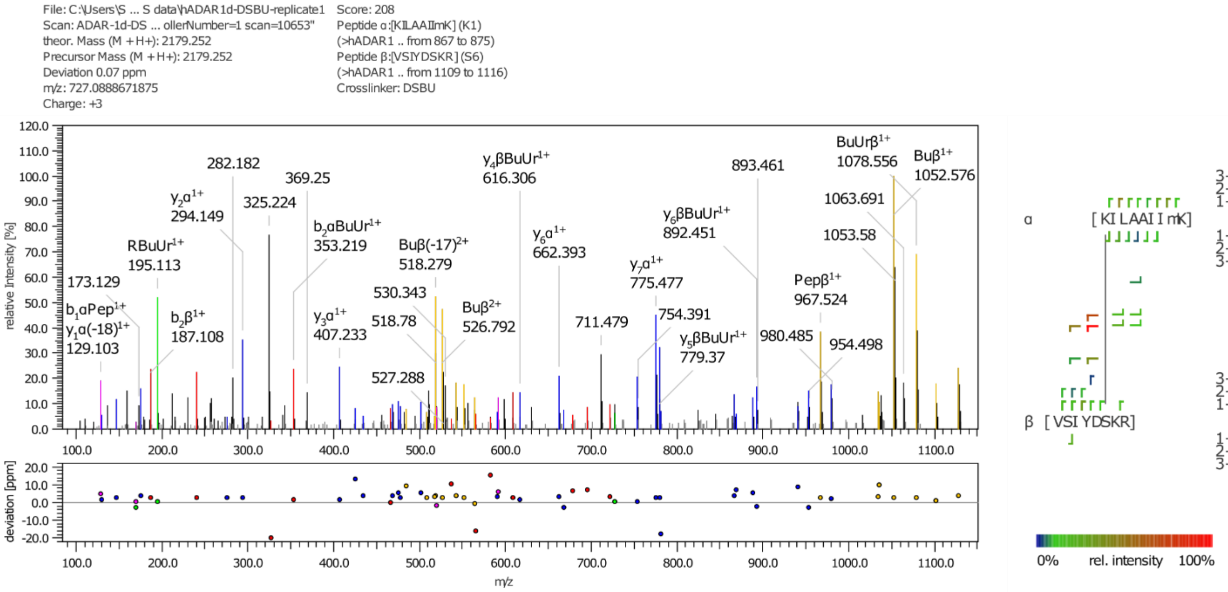

Supplementary Figure 9a. Representative annotated MS2 spectrum of the cross-linked residues (K867 and S1114).

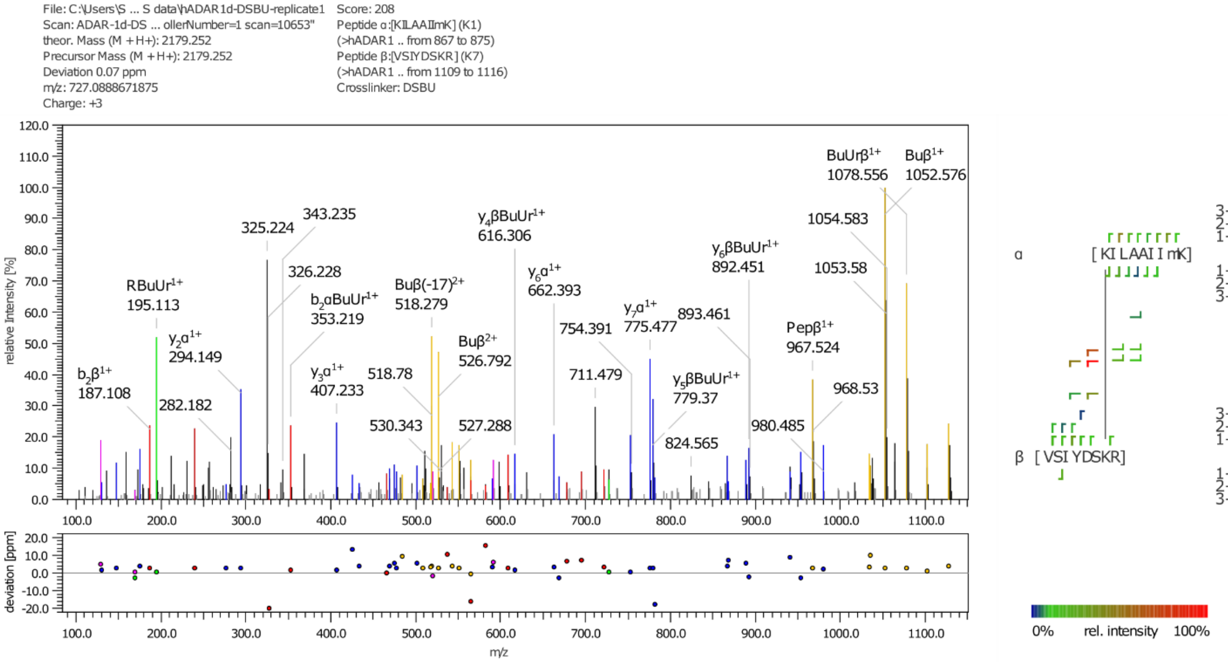

Supplementary Figure 9b. Representative annotated MS2 spectrum of the cross-linked residues (K867 and K1115).

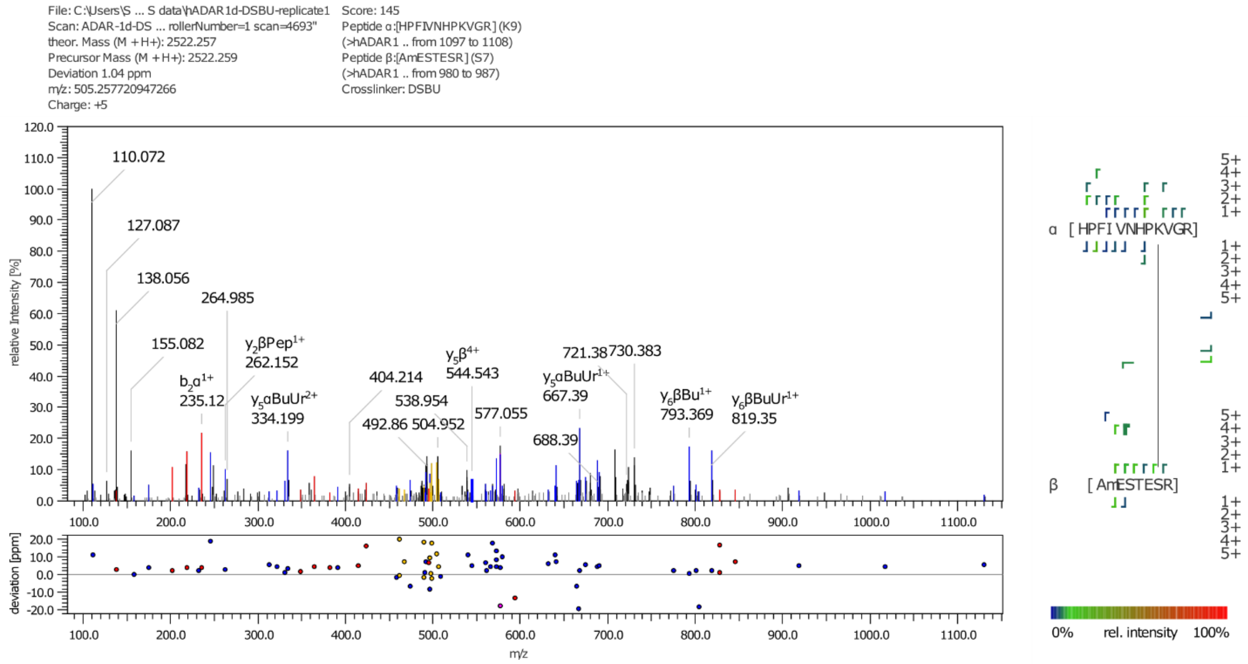

Supplementary Figure 9c. Representative annotated MS2 spectrum of the cross-linked residues (S986 and K1105).

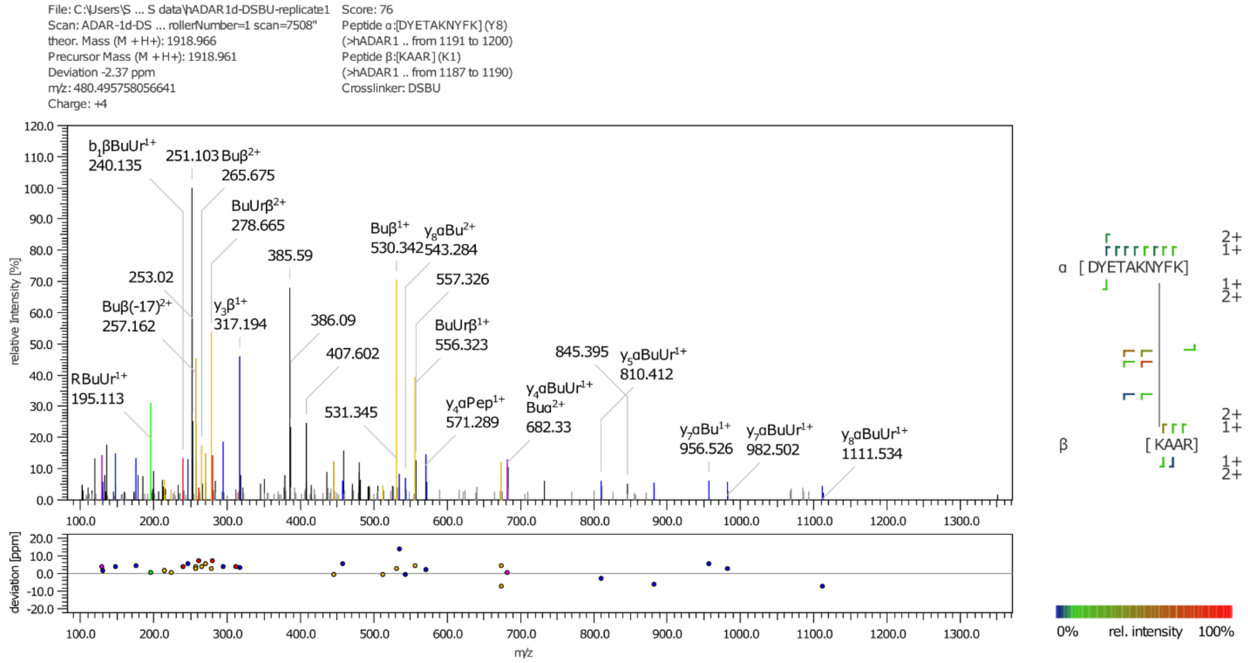

Supplementary Figure 9d. Representative annotated MS2 spectrum of the cross-linked residues (K1187 and Y1198).

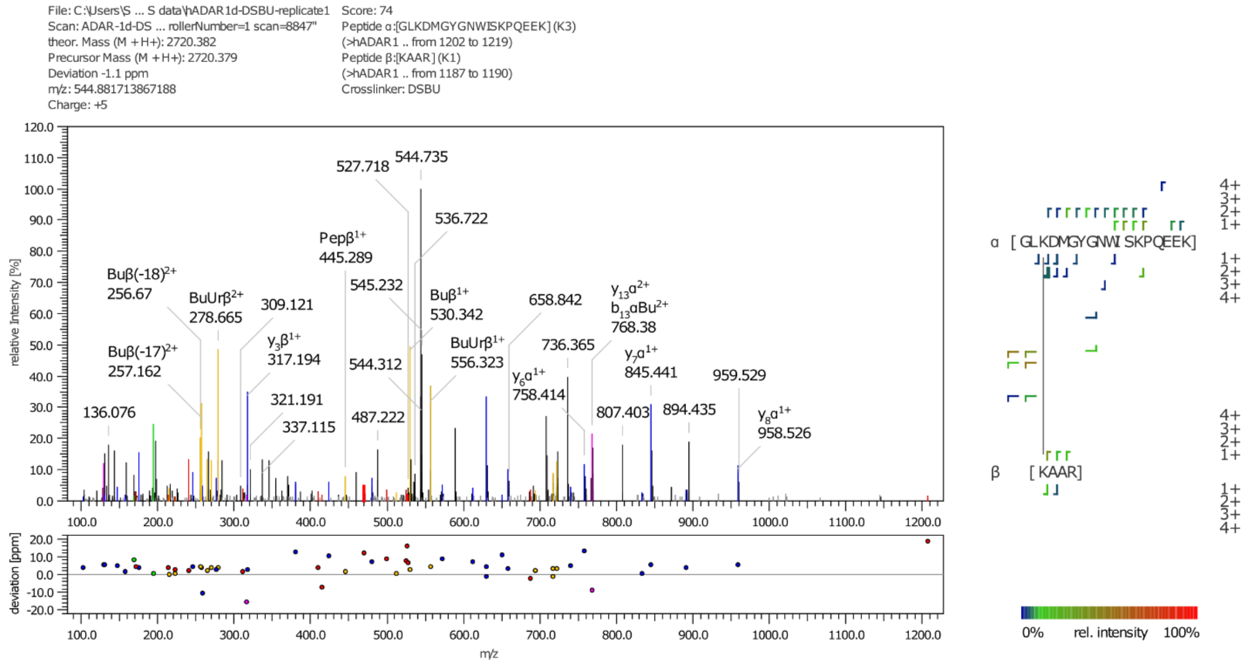

Supplementary Figure 9e. Representative annotated MS2 spectrum of the cross-linked residues (K1187 and K1204).

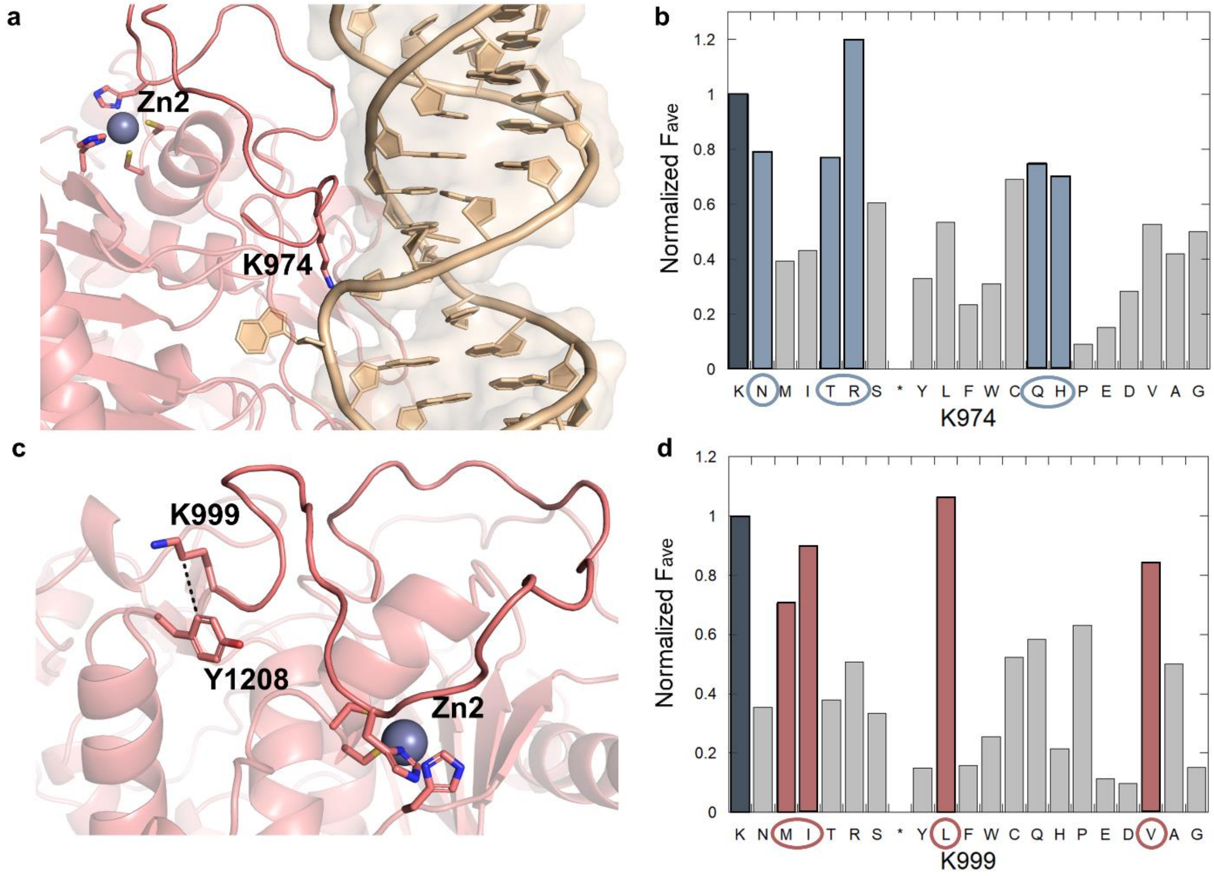

Supplementary Figure 10. Comparison of two lysine residues within the 5' binding loop of hADAR1d. (a) hADAR1d model structure suggests that K974 is a potential RNA contact residue. (b) Previously reported Sat-FACS-Seq data [3] also supports that K974 is an RNA contacting residue because the mutation with polar residues (highlighted in blue) shows a comparable activity to WT. (c) hADAR1d model structure suggesting a hydrophobic interaction between K999 and Y1208, stabilizing the 5' binding loop fold. (d) Sat-FACS-Seq data [3] also supports this by showing a preference of hydrophobic amino acids highlighted in salmon. Thus, one of Aicardi-Goutierres Syndrome (AGS)-associated mutations (K999N) [2] could disrupt the protein structure by introducing a short polar side chain into this hydrophobic site.

5’-
CGGATTAGAAGCCGCCGAGCGGGTGACAGCCCTCCGAAGGAAGACTCTCCTCCGTGCGTCCTCGTCTT
CACCGGTCGCGTTCCTGAAACGCAGATGTGCCTCGCGCCGCACTGCTCCGAACAATAAAGATTCTACA
ATACTAGCTTTTATGGTTATGAAGAGGAAAAATTGGCAGTAACCTGGCCCCACAAACCTTCAAATGAA
CGAATCAAATTAACAACCATAGGATGATAATGCGATTAGTTTTTTAGCCTTATTTCTGGGGTAATTAAT
CAGCGAAGCGATGATTTTTGATCTATTAACAGATATATAAATGCAAAAACTGCATAACCACTTTAACT
AATACTTTCAACATTTTCGGTTTGTATTACTTCTTATTCAAATGTAATAAAAGTATCAACAAAAAATTG
TTAATATACCTCTATACTTTAACGTCAAGGAGAAAAAACCCCGGATCCGTAACCATGTCAAAGATCGA
AGAAGGTAAATTGGTCATCTGGATCAATGGTGATAAGGGTTATAATGGTTTGGCCGAAGTTGGTAAGA
AGTTCGAAAAAGATACTGGTATCAAGGTTACCGTTGAACACCCAGATAAGTTGGAAGAAAAGTTTCCA
CAAGTTGCTGCTACTGGTGATGGTCCAGATATTATCTTTTGGGCTCATGATAGATTTGGTGGTTATGCT
CAATCTGGTTTGTTGGCTGAAATTACTCCAGATAAGGCTTTCCAAGACAAGTTGTATCCTTTTACTTGG
GATGCCGTTAGGTACAACGGTAAATTGATTGCTTATCCAATTGCCGTTGAAGCCTTGTCTTTGATCTAC
AACAAAGACTTGTTGCCCAATCCACCAAAAACCTGGGAAGAAATTCCAGCTTTGGACAAAGAATTGA
AGGCCAAAGGTAAATCCGCTCTGATGTTTAACTTGCAAGAACCATATTTCACCTGGCCATTGATTGCA
GCTGATGGTGGTTACGCTTTTAAGTACGAAAATGGCAAGTACGATATCAAGGATGTTGGTGTTGATAA
TGCTGGTGCTAAAGCTGGTTTGACTTTCTTGGTTGATCTGATCAAAAACAAGCACATGAACGCTGATAC
CGATTACTCTATTGCTGAAGCTGCTTTTAACAAGGGTGAAACTGCTATGACTATTAACGGTCCATGGGC
TTGGTCTAACATTGATACTTCTAAGGTTAACTACGGTGTTACCGTTTTGCCAACTTTTAAAGGTCAACC
ATCTAAGCCATTCGTTGGTGTTTTGTCTGCTGGTATTAACGCTGCTTCTCCAAACAAAGAACTGGCTAA
AGAATTCCTGGAAAACTACTTGTTGACCGACGAAGGTTTGGAAGCTGTTAACAAAGATAAGCCATTGG
GTGCTGTTGCTTTGAAGTCTTATGAAGAAGAATTGGCTAAGGACCCAAGAATTGCTGCAACTATGGAA
AATGCTCAAAAGGGTGAGATTATGCCAAACATCCCACAAATGTCTGCTTTTTGGTATGCTGTTAGAAC
CGCTGTTATCAATGCTGCTTCTGGTAGACAAACTGTTGATGAAGCTTTGAAGGATGCTCAAACCGAGA
ACTTGTATTTCCAGGGATTGCCATTGACTGGTTCTACTTTCCACGACCAAATCGCTATGTTGTCTCACA
GATGTTTCAACACTTTGACTAACTCTTTCCAACCATCTTTGTTGGGTAGAAAGATCTTGGCTGCTATCA
TCATGAAGAAGGACTCTGAAGACATGGGTGTTGTTGTTTCTTTGGGTACTGGTAACAGATGTGTTAAG
GGTGACTCTTTGTCTTTGAAGGGTGAAACTGTTAACGACTGTCACGCTGAAATCATCTCTAGAAGAGG
TTTCATCAGATTCTTGTACTCTGAATTGATGAAGTACAACTCTCAAACTGCTAAGGACTCTATCTTCGA
ACCAGCTAAGGGTGGTGAAAAGTTGCAAATCAAGAAGACTGTTTCTTTCCACTTGTACATCTCTACTGC
TCCATGTGGTGACGGTGCTTTGTTCGACAAGTCTTGTTCTGACAGAGCTATGGAATCTACTGAATCTAG
ACACTACCCAGTTTTCGAAAACCCAAAGCAAGGTAAGTTGAGAACTAAGGTTGAAAACGGTGAAGGT
ACTATCCCAGTTGAATCTTCTGACATCGTTCCAACTTGGGACGGTATCAGATTGGGTGAAAGATTGAG
AACTATGTCTTGTTCTGACAAGATCTTGAGATGGAACGTTTTGGGTTTGCAAGGTGCTTTGTTGACTCA
CTTCTTGCAACCAATCTACTTGAAGTCTGTTACTTTGGGTTACTTGTTCTCTCAAGGTCACTTGACTAGA
GCTATCTGTTGTAGAGTTACTAGAGACGGTTCTGCTTTCGAAGACGGTTTGAGACACCCATTCATCGTT
AACCACCCAAAGGTTGGTAGAGTTTCTATCTACGACTCTAAGAGACAATCTGGTAAGACTAAGGAAAC
TTCTGTTAACTGGTGTTTGGCTGACGGTTACGACTTGGAAATCTTGGACGGTACTAGAGGTACTGTTGA
CGGTCCAAGAAACGAATTGTCTAGAGTTTCTAAGAAGAACATCTTCTTGTTGTTCAAGAAGTTGTGTTC
TTTCAGATACAGAAGAGACTTGTTGAGATTGTCTTACGGTGAAGCTAAGAAGGCTGCTAGAGACTACG
AAACTGCTAAGAACTACTTCAAGAAGGGTTTGAAGGACATGGGTTACGGTAACTGGATCTCTAAGCCA
CAAGAAGAAAAGAACTTCTACTTGTGTCCAGTTTAGCTCGAG -3’

Supplementary Figure 11. Sequence of MBP-hADAR1d WT gene starting from the GAL1 promoter sequence to the end of the gene. Blue: MBP, Green: TEV cleavage site, Red: hADAR1d WT

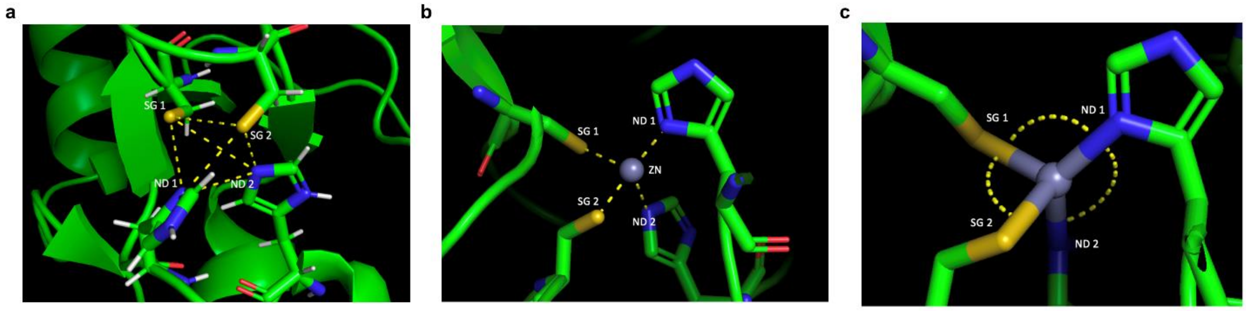

Supplementary Figure 12. Representation of measurement values used as constraints for Rosetta modeling. (a) Distances measured between metal binding residues. (b) Zinc binding distances of metal binding residues. (c) Zinc binding angles of metal binding residues.

Supplementary Table 1. ICP-MS data of zinc ion metal represented as mean ± s.d. from three independent analysis.

Protein	Metal		1	2	3	mean	s.d.
His_{10}-hADAR1d WT	Zn	Metal in ppb	2923.53	2747.82	2750.85		
		Metal in uM	44.71	42.02	42.07		
		Metal/Protein	2.01	1.89	1.89	**1.93**	**0.06**
His_{10}-hADAR2d WT	Zn	Metal in ppb	7020.59	7141.11	7431.58		
		Metal in uM	109.21	109.21	113.65		
		Metal/Protein	0.88	0.90	0.93	**0.90**	**0.02**
MBP-hADAR1d WT	Zn	Metal in ppb	18190.01	18932.90	19447.91		
		Metal in uM	278.18	289.54	297.41		
		Metal/Protein	1.20	1.25	1.28	**1.24**	**0.03**
MBP-hADAR1d C1082D	Zn	Metal in ppb	3528.02	3783.85	3801.00		
		Metal in uM	53.95	57.87	58.13		
		Metal/Protein	0.24	0.26	0.26	**0.251**	**0.008**
MBP-hADAR1d C1082E	Zn	Metal in ppb	1533.22	1565.76	1487.84		
		Metal in uM	23.45	23.94	22.75		
		Metal/Protein	0.18	0.18	0.17	**0.176**	**0.004**
MBP-hADAR1d H1103D	Zn	Metal in ppb	287.94	328.34	281.20		
		Metal in uM	4.40	5.02	4.30		
		Metal/Protein	0.48	0.55	0.47	**0.50**	**0.03**
MBP-hADAR1d H988D	Zn	Metal in ppb	1762.92	1720.45	1724.87		
		Metal in uM	26.96	26.31	26.38		
		Metal/Protein	0.57	0.55	0.55	**0.56**	**0.03**
MBP-hADAR1d C893D	Zn	Metal in ppb	3744.05	3771.33	3654.89		
		Metal in uM	57.26	57.67	55.89		
		Metal/Protein	1.13	1.14	1.10	**1.13**	**0.01**

Supplementary Table 2. Measurements used as constraints for Rosetta modeling. The constraints were defined using average measurements from 20 different PDBs (PDB: 1A1H, 1BBO, 1FRE, 1GUP, 1IA6, 1K6Y, 1LLM, 1ODH, 1RMD, 1SVM, 1WIR, 1WJP, 1X3C, 1X6F, 1X3I, 1UN6, 1V5N, 1YUI, 1ZW8, 2A25) each containing zinc bound by two Cys and two His residues.

(a) Distances measured between metal binding residues.

Atom 1	Atom 2	Average Distance (Å)	Standard Deviation (Å)
Cys SG 1	Cys SG 2	3.81	0.24
Cys SG 2	His ND 2	3.61	0.33
His ND 1	His ND 2	3.47	0.41
His ND 2	Cys SG 1	3.66	0.23

(b) Zinc binding distances of metal binding residues.

Atom 1	Atom 2	Average Distance (Å)	Standard Deviation (Å)
Cys SG	Zn	2.31	0.09
His ND	Zn	2.12	0.13

(c) Zinc binding angles of metal binding residues.

Atom 1	Atom 2	Atom 3	Average Angle (Radians)	Standard Deviation (Radians)
Cys SG 1	Zn	Cys SG 2	1.92	0.16
His ND	Zn	Cys SG	1.86	0.21
His ND 1	Zn	His ND 2	1.94	0.15

Supplementary Table 3. Cross-linked residues identified within hADAR1d WT from all three replicates using DSBU.

Site1	Site2	Cα-Cα (Å)
K867	S1114/K1115	14.5/11.6
S986	K1105	14.6
K1187	Y1198	15.2
K1187	K1204	21.8

Supplementary Table 4. Primers used in saturation mutagenesis to generate hADAR1d cysteine libraries. Bold: NNS codon used for saturation mutagenesis, underline: silent mutation

C851X FWD	5’-CAGATAGCCATGCTGAGCCACCGA**NNS**TTCAACACTCTGACTAACAGC-3’
C851X RVS	5’-GCTGTTAGTCAGAGTGTTGAA**SNN**TCGGTGGCTCAGCATGGCTATCTG-3’
C893X FWD	5’-GTCAGCTTGGGAACAGGGAATCGT**NNS**GTAAAAGGAGATTCTCTCAGC-3’
C893X RVS	5’-GCTGAGAGAATCTCCTTTTAC**SNN**ACGATTCCCTGTTCCCAAGCTGAC 3’
C909X FWD	5’-AAAGGAGAAACTGTCAATGAT**NNS**CATGCAGAAATAATCTCCCGGAGAGGC- 3’
C909X RVS	5’-GCCTCTCCGGGAGATTATTTCTGCATG**SNN**ATCATTGACAGTTTCTCCTTT-3’
C976X FWD	5’-GGCGCCCTCTTTGACAAGTCT**NNS**AGCGACCGTGCTATGGAA-3’
C976X RVS	5’-TTCCATAGCACGGTCGCT**SNN**AGACTTGTCAAAGAGGGCGCC-3’
C1081X FWD	5’-GGGCATCTGACCCGTGCTATA**NNS**TGTCGTGTGACAAGAGATGGGAGT-3’
C1081X RVS	5’-ACTCCCATCTCTTGTCACACGACA**SNN**TATAGCACGGGTCAGATGCCC-3’
C1082X FWD	5’-CATCTGACCCGTGCTATTTGT**NNS**CGTGTGACAAGAGATGGGAGTGCA-3’
C1082X RVS	5’-TGCACTCCCATCTCTTGTCACACG**SNN**ACAAATAGCACGGGTCAGATG-3’
C1129X FWD	5’-AAGGAGACAAGCGTCAACTGG**NNS**CTAGCTGATGGCTATGACCTG-3’
C1129X RVS	5’-CAGGTCATAGCCATCAGCTAG**SNN**CCAGTTGACGCTTGTCTCCTT-3’
C1169X FWD	5’-AACATTTTTCTTCTATTTAAGAAGCTT**NNS**TCCTTCCGTTACCGCAGGGATCTACTG-3’
C1169X RVS	5’-CAGTAGATCCCTGCGGTAACGGAAGGA**SNN**AAGCTTCTTAAATAGAAGAAAAATGTT-3’
C1224X FWD	5’-CAGGAGGAAAAGAACTTTTATCTG**NNS**CCAGTATAGCTCGAGGATCGTAATGAC-3’
C1224X RVS	5’-GTCATTACGATCCTCGAGCTATACTGG**SNN**CAGATAAAAGTTCTTTTCCTCCTG-3’

Supplementary Table 5. Primers used for two-step PCR to prepare Illumina Miseq sequencing samples of hADAR1d cysteine libraries. Amplicon specific sequence is underlined. Index [i5] or [i7] sequence used for 2nd PCR is described in parentheses.

	**1st PCR primers**
C851X 1st FWD	5’ TCGTCGGCAGCGTCAGATGTGTATAAGAGACAGCATGAGAACCTCTATTTCCAGG 3’
C851X 1st RVS	5’ GTCTCGTGGGCTCGGAGATGTGTATAAGAGACAGGTCTTTTTTCATAATGATGGCG 3’
C893X 1st FWD	5’ TCGTCGGCAGCGTCAGATGTGTATAAGAGACAGTTGCTCGGCCGC 3’
C893X 1st RVS	5’ GTCTCGTGGGCTCGGAGATGTGTATAAGAGACAGGAGAAACCTGATGAAGCCTCT 3’
C909X 1st FWD	5’ TCGTCGGCAGCGTCAGATGTGTATAAGAGACAGAAAGACTCTGAGGACATGGGT 3’
C909X 1st RVS	5’ GTCTCGTGGGCTCGGAGATGTGTATAAGAGACAGAGGTTCAAATATACTATCCTTCGCA 3’
C976X 1st FWD	5’ TCGTCGGCAGCGTCAGATGTGTATAAGAGACAGGCTAAGGGAGGAGAAAAGCTC 3’
C976X 1st RVS	5’ GTCTCGTGGGCTCGGAGATGTGTATAAGAGACAGGCCCTGTCCGTTCTCC 3’
C1081X_C1028X 1st FWD	5’ TCGTCGGCAGCGTCAGATGTGTATAAGAGACAGCTGCAAGGGGCACTGT 3’
C1081X_C1028X 1st RVS	5’ GTCTCGTGGGCTCGGAGATGTGTATAAGAGACAGTTTGGAATCATATATGCTGACTCTG
C1129X 1st FWD	5’ TCGTCGGCAGCGTCAGATGTGTATAAGAGACAGCGACATCCCTTTATTGTCAAC 3’
C1129X 1st RVS	5’ GTCTCGTGGGCTCGGAGATGTGTATAAGAGACAGAAAAATGTTCTTTTTGGAGACC 3’
C1169X 1st FWD	5’ TCGTCGGCAGCGTCAGATGTGTATAAGAGACAGCTGGAGATCCTGGACGG 3’
C1169X 1st RVS	5’ GTCTCGTGGGCTCGGAGATGTGTATAAGAGACAGGCCTTTTTTGAAGTAGTTCTTGG 3’
C1224X 1st FWD	5’ TCGTCGGCAGCGTCAGATGTGTATAAGAGACAGCGTGACTACGAGACGGC 3’
C1224X 1st RVS	5’ GTCTCGTGGGCTCGGAGATGTGTATAAGAGACAGCGCGTAATTAAACTTTCTATTATGCT 3’

	**2nd PCR primers**
C851X 2nd FWD	5’ AATGATACGGCGACCACCGAGATCTACACTATCCTCTTCGTCGGCAGCGTC 3’ (N503)
C851X 2nd RVS R1	5’ CAAGCAGAAGACGGCATACGAGATTAAGGCGAGTCTCGTGGGCTCGG 3’ (N701)
C851X 2nd RVS R2	5’ CAAGCAGAAGACGGCATACGAGATCGTACTAGGTCTCGTGGGCTCGG 3’ (N702)
C851X 2nd RVS R3	5’ CAAGCAGAAGACGGCATACGAGATAGGCAGAAGTCTCGTGGGCTCGG 3’(N703)
C851X 2nd RVS R4	5’ CAAGCAGAAGACGGCATACGAGATTCCTGAGCGTCTCGTGGGCTCGG 3’(N704)
C851X 2nd RVS R5	5’ CAAGCAGAAGACGGCATACGAGATGGACTCCTGTCTCGTGGGCTCGG 3’ (N705)
C893X 2nd FWD	5’ AATGATACGGCGACCACCGAGATCTACACTATCCTCTTCGTCGGCAGCGTC 3’ (N503)
C893X 2nd RVS R1	5’ CAAGCAGAAGACGGCATACGAGATTAGGCATGGTCTCGTGGGCTCGG 3’ (N706)

C893X 2nd RVS R2	5’ CAAGCAGAAGACGGCATACGAGATCTCTCTACGTCTCGTGGGCTCGG 3’ (N707)
C893X 2nd RVS R3	5’ CAAGCAGAAGACGGCATACGAGATCAGAGAGGGTCTCGTGGGCTCGG 3’ (N708)
C893X 2nd RVS R4	5’ CAAGCAGAAGACGGCATACGAGATGCTACGCTGTCTCGTGGGCTCGG 3’ (N709)
C893X 2nd RVS R5	5’ CAAGCAGAAGACGGCATACGAGATCGAGGCTGGTCTCGTGGGCTCGG 3’ (N710)
C909X 2nd FWD	5’ AATGATACGGCGACCACCGAGATCTACACAGAGTAGATCGTCGGCAGCGTC 3’ (N504)
C909X 2nd RVS R1	5’ CAAGCAGAAGACGGCATACGAGATTAAGGCGAGTCTCGTGGGCTCGG 3’ (N701)
C909X 2nd RVS R2	5’ CAAGCAGAAGACGGCATACGAGATCGTACTAGGTCTCGTGGGCTCGG 3’ (N702)
C909X 2nd RVS R3	5’ CAAGCAGAAGACGGCATACGAGATAGGCAGAAGTCTCGTGGGCTCGG (N703)
C909X 2nd RVS R4	5’ CAAGCAGAAGACGGCATACGAGATTCCTGAGCGTCTCGTGGGCTCGG (N704)
C909X 2nd RVS R5	5’ CAAGCAGAAGACGGCATACGAGATGGACTCCTGTCTCGTGGGCTCGG (N705)
C976X 2nd FWD	5’ AATGATACGGCGACCACCGAGATCTACACAGAGTAGATCGTCGGCAGCGTC 3’ (N504)
C976X 2nd RVS R1	**5’** CAAGCAGAAGACGGCATACGAGATTAGGCATGGTCTCGTGGGCTCGG 3’ (N706)
C976X 2nd RVS R2	5’ CAAGCAGAAGACGGCATACGAGATCTCTCTACGTCTCGTGGGCTCGG 3’ (N707)
C976X 2nd RVS R3	5’ CAAGCAGAAGACGGCATACGAGATCAGAGAGGGTCTCGTGGGCTCGG 3’ (N708)
C976X 2nd RVS R4	5’ CAAGCAGAAGACGGCATACGAGATGCTACGCTGTCTCGTGGGCTCGG 3’ (N709)
C976X 2nd RVS R5	5’ CAAGCAGAAGACGGCATACGAGATCGAGGCTGGTCTCGTGGGCTCGG 3’ (N710)
C1081X_C1082X 2nd FWD	5’ AATGATACGGCGACCACCGAGATCTACACGTAAGGAGTCGTCGGCAGCGTC 3’ (N505)
C1081X_C1082X 2nd RVS R1	5’ CAAGCAGAAGACGGCATACGAGATTAAGGCGAGTCTCGTGGGCTCGG 3’ (N701)
C1081X_C1082X 2nd RVS R2	5’ CAAGCAGAAGACGGCATACGAGATCGTACTAGGTCTCGTGGGCTCGG 3’ (N702)
C1081X_C1082X 2nd RVS R3	5’ CAAGCAGAAGACGGCATACGAGATAGGCAGAAGTCTCGTGGGCTCGG (N703)
C1081X_C1082X 2nd RVS R4	5’ CAAGCAGAAGACGGCATACGAGATTCCTGAGCGTCTCGTGGGCTCGG (N704)
C1081X_C1082X 2nd RVS R5	5’ CAAGCAGAAGACGGCATACGAGATGGACTCCTGTCTCGTGGGCTCGG (N705)
C1129X 2nd FWD	5’ AATGATACGGCGACCACCGAGATCTACACGTAAGGAGTCGTCGGCAGCGTC 3’ (N505)
C1129X 2nd RVS R1	5’ CAAGCAGAAGACGGCATACGAGATTAGGCATGGTCTCGTGGGCTCGG 3’ (N706)
C1129X 2nd RVS R2	5’ CAAGCAGAAGACGGCATACGAGATCTCTCTACGTCTCGTGGGCTCGG 3’ (N707)
C1129X 2nd RVS R3	5’ CAAGCAGAAGACGGCATACGAGATCAGAGAGGGTCTCGTGGGCTCGG 3’ (N708)
C1129X 2nd RVS R4	5’ CAAGCAGAAGACGGCATACGAGATGCTACGCTGTCTCGTGGGCTCGG 3’ (N709)
C1129X 2nd RVS R5	5’ CAAGCAGAAGACGGCATACGAGATCGAGGCTGGTCTCGTGGGCTCGG 3’ (N710)
C1169X 2nd FWD	5’ AATGATACGGCGACCACCGAGATCTACACACTGCATATCGTCGGCAGCGTC 3’ (N506)

C1169X 2nd RVS R1	5’ CAAGCAGAAGACGGCATACGAGATTAAGGCGAGTCTCGTGGGCTCGG 3’ (N701)
C1169X 2nd RVS R2	5’ CAAGCAGAAGACGGCATACGAGATCGTACTAGGTCTCGTGGGCTCGG 3’ (N702)
C1169X 2nd RVS R3	5’ CAAGCAGAAGACGGCATACGAGATAGGCAGAAGTCTCGTGGGCTCGG (N703)
C1169X 2nd RVS R4	5’ CAAGCAGAAGACGGCATACGAGATTCCTGAGCGTCTCGTGGGCTCGG (N704)
C1169X 2nd RVS R5	5’ CAAGCAGAAGACGGCATACGAGATGGACTCCTGTCTCGTGGGCTCGG (N705)
C1224X 2nd FWD	5’ AATGATACGGCGACCACCGAGATCTACACACTGCATATCGTCGGCAGCGTC 3’ (N506)
C1224X 2nd RVS R1	5’ CAAGCAGAAGACGGCATACGAGATTAGGCATGGTCTCGTGGGCTCGG 3’ (N706)
C1224X 2nd RVS R2	5’ CAAGCAGAAGACGGCATACGAGATCTCTCTACGTCTCGTGGGCTCGG 3’ (N707)
C1224X 2nd RVS R3	5’ CAAGCAGAAGACGGCATACGAGATCAGAGAGGGTCTCGTGGGCTCGG 3’ (N708)
C1224X 2nd RVS R4	5’ CAAGCAGAAGACGGCATACGAGATGCTACGCTGTCTCGTGGGCTCGG 3’ (N709)
C1224X 2nd RVS R5	5’ CAAGCAGAAGACGGCATACGAGATCGAGGCTGGTCTCGTGGGCTCGG 3’ (N710)

Supplementary Table 6. Primers used for cloning of MBP-tagged hADAR1d WT and for site-directed mutagenesis to prepare metal binding mutants (MBP-hADAR1d C1082D, C1082E, H1103D, H988D, and hADAR1 p110 C1082D, C1082E).

MBP clone FWD	5' AACGTCAAGGAGAAAAAACCCCGGATCCGTAACCATGTCAAAGATCG 3'
MBP clone RVS	5' TCCCTGGAAATACAAGTTCTCGGT 3'
ADAR1 clone FWD	5' GAGAACTTGTATTTCCAGGGATTGCCA 3'
ADAR1 clone RVS	5' ATATAATCTAGTCATTACGATCCTCGAGCTAAACTGGACACAAGTAGAAG 3'
C1082D FWD	5' CACTTGACTAGAGCTATCTGTGATAGAGTTACTAGAGACGGTTCTGCT 3'
C1082D RVS	5' AGCAGAACCGTCTCTAGTAACTCTATCACAGATAGCTCTAGTCAAGTG 3'
C1082E FWD	5' CACTTGACTAGAGCTATCTGTGAAAGAGTTACTAGAGACGGTTCTGCT 3'
C1082E RVS	5' AGCAGAACCGTCTCTAGTAACTCTTTCACAGATAGCTCTAGTCAAGTG 3'
H1103D FWD	5' TTGAGACACCCATTCATCGTTAACGATCCAAAGGTTGGTAGAGTTTCTATC 3'
H1103D RVS	5' GATAGAAACTCTACCAACCTTTGGATCGTTAACGATGAATGGGTGTCTCAA 3'
H998D FWD	5' GCTATGGAATCTACTGAATCTAGAGATTACCCAGTTTTCGAAAACCCAAAG 3'
H988D RVS	5' CTTTGGGTTTTCGAAAACTGGGTAATCTCTAGATTCAGTAGATTCCATAGC 3'
A1 p110 C1082D FWD	5' CATCTGACCCGTGCTATTTGCGACCGTGTGACAAGAGATGGGAGT 3'
A1 p110 C1082D RVS	5' ACTCCCATCTCTTGTCACACGGTCGCAAATAGCACGGGTCAGATG 3'
A1 p110 C1082E FWD	5' CATCTGACCCGTGCTATTTGCGAGCGTGTGACAAGAGATGGGAGT 3'
A1 p110 C1082E RVS	5' ACTCCCATCTCTTGTCACACGCTCGCAAATAGCACGGGTCAGATG 3'

Supplementary Table 7. Primers used in RT-PCR to confirm *in vitro* deamination assay on hGli1 RNA. hGLI1 150 RT FWD primer was also used for Sanger sequencing to confirm the editing level.

hGLI1 150 RT FWD	5'-CAGAACTTTGATCCTTACCTCC-3'
hGLI1 150 RT RVS	5'-CATATAGGGGTTCAGACCACTG-3'

Supplementary Table 8. Primers used in RT-PCR and Nested PCR to confirm the editing level of the endogenous editing sites in HEK293T cells. Either Nest FWD or Nest RVS primer of each endogenous site was used for Sanger sequencing to confirm the editing level.

AZIN1 RT FWD	5'-GAAGGATCTGGTGTTAAGATAATTTCAGAACCCG-3'
AZIN1 RT RVS	5'-ACTGGAATGTTGACCAGACAAGCTTAACC-3'
AZIN1 Nest FWD	5'-GAAGCTACTATGTGTCTTCTGCATTTACAC-3'
AZIN1 Nest RVS	5'-TGCAACTTCAGATCTAAAGAAGCGT-3'
COG3 RT FWD	5'-CAGATGCATAGATAGGGCAGTGTTCCAAGGA-3'
COG3 RT RVS	5'-ACCTTTGTCATGAACTCCTCCAGCTGTTC-3'
COG3 Nest FWD	5'-TTATCACAGGAAGCATTGTCTGCCTGCATTCAGTC-3'
COG3 Nest RVS	5'-TACAAACAGCTTGGTCTGCTGCTGAAT-3'
NUP43 RT FWD	5'-TCTTTCCAAACTTTGTTAGATTTTAAATGTATTATTGACCTGAGA-3'
NUP43 RT RVS	5'-CGGTGTTACATTACATAAAGCTTAGTTTCTTATTAAAATTGGCAA-3'
NUP43 Nest FWD	5'-CCTTAATGACAAATCACTGCTATTAGACAATTG-3'
NUP43 Nest RVS	5'-AACTTGTCAATTGGCATGAAATGTAATGCT-3'
GLI1 RT FWD	5'-CGAGCCGAGTATCCAGGATACAAC-3'
GLI1 RT RVS	5'-CCCATATCCCAGAGTATCAGTAGGTGG-3'
GLI1 Nest FWD	5'-CCCAATGCAGGGGTCACCCGGAGGG-3'
GLI1 Nest RVS	5'-GAAGTCCATATAGGGGTTCAGACCACTGCCCAC-3'

Supplementary methods

Fluorescence based activity assay. Mutations in hADAR1d H1103 were introduced by QuikChange II XL site-directed mutagenesis kit (Agilent) following manufacturer's protocol. Florescent reporter plasmid containing BDF2 substrate sequence and each of mutant hADAR1d expression plasmids were sequentially transformed into yeast INVSc1 strain using TRP1 and URA3 selection, respectively. Then, yeast cells were plated on selection plate (CM – ura – trp + 2% glucose) for growth. From a selection plate, a single colony was inoculated to a 5 mL CM – ura – trp + 2% glucose media for overnight growth. The resulting culture of 0.1 ml was used to inoculate a 20 ml CM – ura -trap + 3% glycerol + 2% lactate media for further growth until OD_{600} of the culture reaches 1-2 (approximately 30 h). For induction, 30% galactose was added to each culture to a final concentration of 3% followed by harvesting after approximately 10 h of induction. The same number of cells were pelleted, washed twice with PBS and used to measure the fluorescence intensity in an Optiplate-96 black, black opaque 96-well microplate (PerkinElmer) using a CLARIOstar plate reader (BMG labtech), with excitation at 482/16 nm and emission at 520/10 nm. The fluorescence readings were normalized to the reading of hADAR1d WT for F/F_{WT} value, which then used for a bar graph generated by Graphpad Prism 8. The experiments were performed in biological triplicate.

Preparation of hGli1 RNA for *in vitro* deamination assay. hGli1 RNA (147 nt) was prepared by *in vitro* transcription and purified by a denaturing polyacrylamide gel as reported previously [5]. In brief, a truncated 147 nt hGli1 sequence containing plasmid (pYES/CT vector) was used to amplify a fragment containing T7 promotor and 147 nt hGli1 sequence, after which *in vitro* RNA transcription was carried out using T7 MEGAscript kit (Life techonologies). Transcribed RNAs were purified using a denaturing polyacrylamide gel as described previously [5] and refolded to the desired concentration in annealing buffer (100 mM NaCl, 10 mM Tris-HCl, pH 8.0, 1 mM EDTA) by heating at 95 °C for 5 min and then slowly cooled to room temperature. Solutions of refolded RNAs were stored at -20 °C and used for deamination assays.

Detection of hADAR1 p110 proteins in HEK293T cells. Western blotting of hADAR1 p110 proteins in HEK293T cells was carried out as previously described [6] to detect the expression of each protein. In brief, cells were lysed with 300 μL of lysis buffer (50 mM Tris-HCl, pH 8.0, 150 mM NaCl, 1%(v/v) NP-40 supplemented with Halt protease inhibitor cocktail) (Thermo Fisher Scientific) by shaking at 4 °C for 30 min. Each sample was resolved on an SDS-PAGE and western blotting was performed using primary antibody HA Tag Monoclonal Antibody (2-2.2.14) (Thermo Fisher Scientific) at 1:10,000 dilution and anti-mouse IgG with alkaline phosphatase-conjugated secondary antibody (Santa Cruz Biotechnology) at 1:2,000 dilution. The hADAR1 p110 proteins were detected using ECF substrate (GE Healthcare) on a Typhoon Trio Variable Mode Imager (GE Healthcare) (**Supplementary Figure 3**).

Supplementary references

1. Matthews, M. M. *et al.* Structures of human ADAR2 bound to dsRNA reveal base-flipping mechanism and basis for site selectivity. *Nat. Struct. Mol. Biol.* **23**, 426–433 (2016).
2. Fisher, A. J. & Beal, P. A. Effects of Aicardi-Goutières syndrome mutations predicted from ADAR-RNA structures. *RNA Biol.* **14**, 164–170 (2017).
3. Wang, Y., Park, S. & Beal, P. A. Selective Recognition of RNA Substrates by ADAR Deaminase Domains. *Biochemistry* **57**, 1640–1651 (2018).
4. Iacobucci, C. *et al.* A cross-linking/mass spectrometry workflow based on MS-cleavable cross-linkers and the MeroX software for studying protein structures and protein–protein interactions. *Nat. Protoc.* **13**, 2864–2889 (2018).
5. Eifler, T., Pokharel, S. & Beal, P. A. RNA-Seq analysis identifies a novel set of editing substrates for human ADAR2 present in Saccharomyces cerevisiae. *Biochemistry* **52**, 7857–7869 (2013).
6. Monteleone, L. R. *et al.* A Bump-Hole Approach for Directed RNA Editing. *Cell Chem. Biol.* **26**, 269-277.e5 (2019).
