# Supplementary material for: High-throughput mutagenesis reveals unique structural features of human ADAR1
Source: Nat Commun. 2020 Oct 12;11:5130. doi: 10.1038/s41467-020-18862-2 (PMC7550611; doi:10.1038/s41467-020-18862-2)
Supplement: Supplementary file 3 — Reporting Summary [file 41467_2020_18862_MOESM3_ESM.pdf]

nature research

Corresponding author(s): Peter A. Beal

Last updated by author(s): 09-01-2020

Reporting Summary

Nature Research wishes to improve the reproducibility of the work that we publish. This form provides structure for consistency and transparency in reporting. For further information on Nature Research policies, see Authors & Referees and the Editorial Policy Checklist.

Statistics

For all statistical analyses, confirm that the following items are present in the figure legend, table legend, main text, or Methods section.

n/a	Confirmed	
☐	☒	The exact sample size (n) for each experimental group/condition, given as a discrete number and unit of measurement
☐	☒	A statement on whether measurements were taken from distinct samples or whether the same sample was measured repeatedly
☒	☐	The statistical test(s) used AND whether they are one- or two-sided<br>*Only common tests should be described solely by name; describe more complex techniques in the Methods section.*
☒	☐	A description of all covariates tested
☒	☐	A description of any assumptions or corrections, such as tests of normality and adjustment for multiple comparisons
☐	☒	A full description of the statistical parameters including central tendency (e.g. means) or other basic estimates (e.g. regression coefficient) AND variation (e.g. standard deviation) or associated estimates of uncertainty (e.g. confidence intervals)
☒	☐	For null hypothesis testing, the test statistic (e.g. F, t, r) with confidence intervals, effect sizes, degrees of freedom and P value noted<br>*Give P values as exact values whenever suitable.*
☒	☐	For Bayesian analysis, information on the choice of priors and Markov chain Monte Carlo settings
☒	☐	For hierarchical and complex designs, identification of the appropriate level for tests and full reporting of outcomes
☒	☐	Estimates of effect sizes (e.g. Cohen's d, Pearson's r), indicating how they were calculated

Our web collection on statistics for biologists contains articles on many of the points above.

Software and code

Policy information about availability of computer code

Data collection	FlowJo v10 was used to collect the cell sorting data. Promals3D and Rosetta (2014 version) were used for a homology modeling of hADAR1 catalytic domain structure.
Data analysis	FlowJo v10 was used to analyze the cell sorting data and generate a contour plot. Trimmomatic v0.39, FastQC v0.11.9, and MobaXterm v10.4 were used to analyze the Miseq data. Seq2 Logo 2.0 server was utilized to obtain a logoplot of Sat-FACS-Seq analysis. All graphs were generated using either KaleidaGraph v4.0, or GraphPad Prism 8. Chromas v2.6.6 was used to obtain the editing level based on sequencing traces. SWISS-MODEL web server and Metal Ion-Binding Site Prediction and Docking Server (MIB) were used to predict a second metal binding site. Microsoft Excel was used to analyze Sat-FACS-Seq data and ICP-MS data. Protein native MS data was analyzed by Agilent MassHunter software (B.07.00 Service Pack 2 Build 7.07024.29) and ICP-MS data was analyzed by Agilent MassHunter ICP-MS software (G7201C, Version C.01.03). pXtract v2.0 and MeroX v2.0 were used for chemical crosslink MS analysis.

For manuscripts utilizing custom algorithms or software that are central to the research but not yet described in published literature, software must be made available to editors/reviewers. We strongly encourage code deposition in a community repository (e.g. GitHub). See the Nature Research guidelines for submitting code & software for further information.

Data

Policy information about availability of data

All manuscripts must include a data availability statement. This statement should provide the following information, where applicable:

- Accession codes, unique identifiers, or web links for publicly available datasets
- A list of figures that have associated raw data
- A description of any restrictions on data availability

All data supporting the findings of this study are available within the article and its supplementary information files. The source data underlying Figs 4d, 6b-c and Supplementary Figure 3 are provided as a Source Data file. Illumina sequencing data are available in the NCBI Sequence Read Archive (SRA) under accession code PRJNA590991 [https://www.ncbi.nlm.nih.gov/bioproject/PRJNA590991/]. The cross-linking mass spectrometry (XL-MS) data, and intact and native mass spectrometry data have been deposited to the Proteome Xchange Consortium via the MassIVE partner repository with the data set identifier PXD021052 [https://

doi.org/doi:10.25345/C5T758] and PXD021175 [https://doi.org/doi:10.25345/C5VV0W], respectively. All input files and the lowest 10 energy structures of ADAR1d are available in GitHub at https://github.com/siegel-lab-ucd/Publication_Tiffy/tree/master/High-throughput%20Mutagenesis%20Reveals%20Unique%20Structural%20Features%20of%20Human%20ADAR1.

Field-specific reporting

Please select the one below that is the best fit for your research. If you are not sure, read the appropriate sections before making your selection.

☒ Life sciences ☐ Behavioural & social sciences ☐ Ecological, evolutionary & environmental sciences

For a reference copy of the document with all sections, see nature.com/documents/nr-reporting-summary-flat.pdf

Life sciences study design

All studies must disclose on these points even when the disclosure is negative.

Sample size	All sample size was described either in the Method section or in the figure legends. Sample sizes were chosen based on the previous publications and literatures.
Data exclusions	All data was included in the study.
Replication	In vitro deamination kinetics of MBP-hADAR1d WT and metal binding mutants was carried out in technical triplicate and the reproducibility was confirmed. Endogenous editing in HEK293T cells as well as fluorescence based cell activity assay were performed in biological triplicates and confirmed that it could be reproducible. ICP-MS of each protein sample was analyzed in technical triplicate and confirmed the reproducibility. Chemical cross-linking using DSBU were performed in three replicates which was reproducible each time.
Randomization	We did not perform any experiments that require randomization.
Blinding	We did not perform any experiments that require blinding.

Reporting for specific materials, systems and methods

We require information from authors about some types of materials, experimental systems and methods used in many studies. Here, indicate whether each material, system or method listed is relevant to your study. If you are not sure if a list item applies to your research, read the appropriate section before selecting a response.

Materials & experimental systems

n/a	Involved in the study
☐	☒ Antibodies
☐	☒ Eukaryotic cell lines
☒	☐ Palaeontology
☒	☐ Animals and other organisms
☒	☐ Human research participants
☒	☐ Clinical data

Methods

n/a	Involved in the study
☒	☐ ChIP-seq
☐	☒ Flow cytometry
☒	☐ MRI-based neuroimaging

Antibodies

Antibodies used	HA Tag Monoclonal Antibody (2-2.2.14), ThermoFisher, Catalog # 26183, AB_10978021; Anti-mouse IgG with alkaline phosphatase-conjugated antibody, Santa Cruz Biotechnology, Catalog # SC2008
Validation	HA Tag Monoclonal Antibody (2-2.2.14): Validation for various applications can be found from the manufacture's website. Anti-mouse IgG with alkaline phosphatase-conjugated antibody: Validation for various applications can be found from the manufacture's website.

Eukaryotic cell lines

Policy information about cell lines

Cell line source(s)	Human: Embryonic Kidney 293T cells, ATCC (CRL-11268; RRID: CVCL_1926)
Authentication	Cell line was not authenticated.
Mycoplasma contamination	Cell line was not tested for mycoplasma contamination.

Commonly misidentified lines
(See ICLAC register)

None was listed as commonly misidentified cell line.

Flow Cytometry

Plots

Confirm that:

☒ The axis labels state the marker and fluorochrome used (e.g. CD4-FITC).

☒ The axis scales are clearly visible. Include numbers along axes only for bottom left plot of group (a 'group' is an analysis of identical markers).

☒ All plots are contour plots with outliers or pseudocolor plots.

☒ A numerical value for number of cells or percentage (with statistics) is provided.

Methodology

Sample preparation

hADAR1d cysteine libraries in hADAR1d E1008Q (in YEpTOP2P-GAL1 vector) was prepared by saturation mutagenesis and transformed into S. cerevisiae yeast cells along with the fluorescent reporter plasmid. Yeast cells were diluted in PBS to 20,000 cells/µl.

Instrument

Beckman Coulter Astrios EQ cell sorter.

Software

FlowJo V10.

Cell population abundance

A relative cell abundance in each gate is R1 35.44%, R2 7.05%, R3 7.41%, R4 14.13%, and R5 12.52%. Cells were sorted using a stringent purity criteria that charges and deflects only droplets in which the green cell of interest is predicted to be isolated from nearby, unwanted events in the saline stream. The Astrios's advanced pulse processing electronics are able to predict the exact position of the cell within the fluid stream and candidate droplet to 1/100th of a drop volume. The Astrios's jet-in-air nozzle configuration enhances stream stability, droplet formation and droplet collection. The sort conditions of the cell sorter and the fluorescence characteristics of the sample were monitored for stability by the operator throughout the experiment to ensure the purity of the individual samples.

Gating strategy

The cells were sorted using the Beckman Coulter Astrios EQ Cell sorter into five different gates (R1-R5) based on different fluorescence levels using a 70 um nozzle at 60 psi fluidic pressure with GFP excitation at 488 nm and emission at 529/28 nm. A background fluorescence was gated first as R1 with cells expressing an inactive mutant of hADAR1d E912A and R2-R5 gates were determined with cells having a fluorescence level above background (GFP intensity ranging from dull (Median fluorescent intensity 200 units), moderate (MFI = 500 units), moderately bright (MFI = 2000 units) and bright (MFI = 5000 units)). Sorting decisions based on regions drawn to encompass cells exhibiting these average fluorescent values were drawn and used to separate cells based on increasing fluorescence intensity.

☒ Tick this box to confirm that a figure exemplifying the gating strategy is provided in the Supplementary Information.
