# Supplementary material for: High-throughput mutagenesis reveals unique structural features of human ADAR1
Source: Nat Commun. 2020 Oct 12;11:5130. doi: 10.1038/s41467-020-18862-2 (PMC7550611; doi:10.1038/s41467-020-18862-2)
Supplement: Supplementary file 4 — Description of Supplementary Data [file 41467_2020_18862_MOESM4_ESM.pdf]

Description of Additional Supplementary Files

File Name: Supplementary Data 1
Description: Abundance and enrichment levels of R1-R5 gates and corresponding F_{ave} values for amino acids at each position of variation in hADAR1d Cys library. **Column A-B**: Position under analysis, randomized codons and the corresponding amino acids introduced to each position. **Column C-H**: Abundance (the number of reads from Illumina sequencing) of each 20 amino acids and the stop codon in each input library and gate R1 through R5. **Column I-N**: Corrected abundance of each 20 amino acids and the stop codon based on the total reads from Illumina sequencing in each input library and gate R1 through R5. **Column O-S**: The enrichment levels in each gate R1 through R5 that was weighted against their abundance in the input library. **Column T**: Average fluorescence values as an indication of relative activities calculated using the enrichment levels of amino acids across the all five gates (R1-R5) and the median fluorescence values of populations in each gate.

File Name: Supplementary Data 2
Description: ICP-MS metal screening data of MBP-hADAR1d WT purified without metal supplementation. Total of 30 metal ions were screened and values are represented as mean ± s.d. from three independent analysis.

File Name: Supplementary Data 3
Description: Cross-link MS data for hADAR1d WT as .mgf file format.
